# Reemergence of Recombinant Vaccine–derived Polioviruses in Healthy Children, Madagascar

**DOI:** 10.3201/eid1906.130080

**Published:** 2013-06

**Authors:** Richter Razafindratsimandresy, Marie-Line Joffret, Sendraharimanana Rabemanantsoa, Seta Andriamamonjy, Jean-Michel Heraud, Francis Delpeyroux

**Affiliations:** Institut Pasteur, Antananarivo, Republic of Madagascar (R. Razafindratsimandresy, S. Rabemanantsoa, S. Andriamamonjy, J-M. Heraud);; Institut Pasteur, Paris, France (M.-L. Joffret, F. Delpeyroux);; Institut National de la Santé et de la Recherche Médicale, Paris (M-L. Joffret, F. Delpeyroux)

**Keywords:** poliovirus, vaccine-derived polioviruses, recombinant, Madagascar, viruses, reemergence, vaccine coverage

**To the Editor:** Poliomyelitis outbreaks caused by pathogenic vaccine-derived polioviruses (VDPVs) are primarily a result of low polio vaccine coverage. Low coverage enables interhuman circulation of polioviruses (PVs) from the oral polio vaccine (OPV), and it enables genetic drift of the viruses and their subsequent reversion to neurovirulent phenotypes (*1*). Polio outbreaks associated with type 2 or 3 VDPVs (VDPV2s or VDPV3s) were reported in 2001–2002 and 2005 in Toliara Province in southern Madagascar (*2*,*3*). These VDPVs were found in patients with acute flaccid paralysis (AFP) and in healthy children who were contacts of the patients with AFP (*2*,*4*). The genomes of these VDPVs belong to several independent, complex mosaic recombinant lineages composed of sequences derived from vaccine polioviruses and other co-circulating species C human enteroviruses (human EV-C) (*4*,*5*). The 2001–2002 and 2005 outbreaks in Toliara Province were stopped after rapid and efficient OPV vaccination campaigns.

No polio cases have been detected in Madagascar since 2005. However, OPV coverage fluctuates in Toliara Province. In June 2011, to determine if VDPVs were circulating in the province, we collected fecal samples from 616 healthy residents <5 years of age (Madagascar Ethics Committee agreement 011-MSANP/CE). Sample extracts were used to inoculate human RD and HEp-2c cells and mouse L cells expressing the human poliovirus cellular receptor CD155 (L20B cells) (*6*). Of the 616 samples, 238 induced cytopathogenic effects in human cells, of which 20 also induced cytopathogenic effects in L20B cells; the latter samples were confirmed by reverse transcription PCR molecular testing (*7*) to contain PV strains. All PV isolates originated from children who had not received OPV in the 30 days before samples were collected. 

We sequenced the genomic regions encoding the capsid viral protein (VP1) of the 20 isolates confirmed to contain PV strains: 3 type 2 PV isolates showed >0.5% nt sequence divergence from the type 2 OPV strain (Sabin 2) and were thus identified as potentially pathogenic VDPV2s. Two of these VDPV2s carried numerous mutations in the VP1 region (903 bp): isolates MAD-2593–11 (30 nt mutations) and MAD-2642–11 (33 nt mutations). The third VDPV2, MAD-2675–11, carried 6 nt mutations. The nucleotide differences between Sabin 2 and MAD-2593–11 (3.3%) and MAD-2642–11 (3.6%) suggest that these VDPV2s had been multiplying or circulating for ≈3.0 years, and the differences between Sabin 2 and MAD-2675–11 suggest it had been multiplying or circulating for ≈0.5 year. Almost complete genomic sequencing, from nt 36 to nt 7,420 (Sabin 2 numbering), was subsequently performed (EMBL accession nos. HF913426– HF913428). 

Isolate MAD-2675–11 was shown to be a mutated Sabin 2 PV, but isolates MAD-2593–11 and MAD-2642–11 displayed mosaic recombinant genomes composed of sequences derived from Sabin 2 and other human EV-Cs. Both recombinants had the 5′–untranslated region (UTR), the nonstructural P2–P3 regions, and the 3′-UTR derived from non-PV human EV-C. The 2 recombination sites were similarly located in each of these recombinant genomes (approximately at nt 760 and nt 3,368) at the 2 extremities of the structural genomic P1 region. Although these recombinants appeared to be related, they had different non-PV human EV-C sequences in the P2–P3 region and the 3′-UTR (6.0%–20.0% nt sequence difference). The phylogenetic relationship of these isolates to the previous Madagascar VDPV2s was assessed: the 2011 isolates originated from 2 novel, independent events (Figure). The 2011 VDPVs had lost the major attenuating determinants in the 5′-UTR (guanine to adenine at nt 481) and VP1 region (isoleucine to threonine at codon 143) regions (*8*). This finding strongly suggests that the 2011 VDPVs had regained a degree of neurovirulence.

**Figure:**
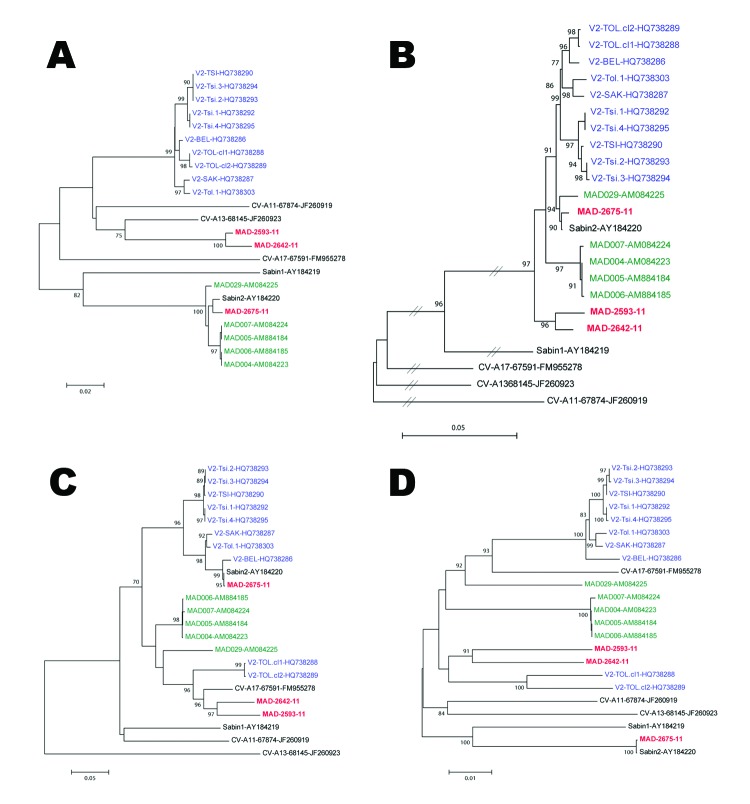
Phylogenetic trees showing genetic relationships between sequences of vaccine-derived poliovirus (VDPV) isolates. The trees are based on nucleotide sequence alignments of various subgenomic regions. Multiple sequence alignments were performed with CLC Main Workbench 5.7.2 software (CLC bio, Aarhus, Denmark). Phylograms were constructed with MEGA 4 (http://megasoftware.net/mega4/mega.html), using the Jukes-Cantor algorithm for genetic distance determination and the neighbor-joining method. The robustness of the resulting trees was assessed with 1,000 bootstrap replications. A) Tree built from 712-bp fragments of the 5′–untranslated region (nt 36–747, with reference to Sabin 2 nucleotide numbering). B) Tree built from 2,637-bp fragments of the P1 region (nt 748–3,384). C) Tree built from 1,725-bp fragments of the P2 region (nt 3,385–5,109). D) Tree built from 2,259-bp fragments of the P3 region (nt 5,110–7,368). Names (isolate name-accession no.) are VDPV isolates recovered in Madagascar in 2011, 2005, and 2001–2002. Sequences of other isolates (CV-A11, 13, and 17) from Madagascar were also used. The percentage of bootstrap replicates is indicated at nodes if >70%. The length of branches is proportional to the number of nucleotide differences (percent divergence).Scale bars indicate genetic distances.

Although there is currently no evidence of AFP cases linked to these new VDPVs, their detection in 3 children and their genetic characteristics strongly suggest insufficient OPV coverage in Toliara Province. We could obtain proof of vaccination (vaccination card) for only 27% of the participants. To prevent a third poliomyelitis outbreak, the Ministry of Health of Madagascar organized house-to-house OPV vaccination campaigns within Toliara Province in October and December 2011 and in January 2012. No polio cases have been reported in the province since then, suggesting that VDPV circulation was limited or stopped by these campaigns.

Massive OPV immunization campaigns worldwide have greatly decreased the frequency of poliomyelitis caused by wild-type PVs. However, after the disease has been eliminated in developing countries, low polio vaccine coverage frequently enables the emergence of VDPVs or the reintroduction of wild-type PV from disease-endemic countries; both scenarios threaten the success of the poliomyelitis eradication program (*9*,*10*). More than 640 polio cases caused by circulating VDPVs have been reported in 21 developing countries. We have shown that even in the absence of polio cases, potentially pathogenic circulating VDPVs can be detected in fecal samples collected from children living in areas with fluctuating and often low polio vaccine coverage. Thus, such sampling can help determine whether vaccine campaigns should be implemented in areas where polio could reemerge. In Madagascar, vaccination appears to have cleared emerging VDPVs and to have prevented polio cases in children.
